# Nutrient pattern analysis of mineral based, simple sugar based, and fat based diets and risk of metabolic syndrome: a comparative nutrient panel

**DOI:** 10.1186/s12902-022-00963-2

**Published:** 2022-03-02

**Authors:** Leila Nikniaz, Trias Mahmudiono, Saade Abdalkareem Jasim, Mahdi Vajdi, Lakshmi Thangavelu, Mahdieh Abbasalizad Farhangi

**Affiliations:** 1 Tabriz Health Services Management Research Center, Tabriz University of Medical Sciences, Tabriz, Iran; 2 Department of Nutrition, Faculty of Public Health, Universitas Airlangga, Kota Surabaya, Indonesia; 3 Medical Laboratory Techniques Department, Al-Maarif University College, Al-anbar-Ramadi, Iraq; 4 Department of Community Nutrition, Faculty of Nutrition, Tabriz University of Medical Sciences, Tabriz, Iran; 5 Department of Pharmacology, Saveetha Institute of Medical and Technical Science, Saveetha Dental College, Saveetha University, Chennai, India

**Keywords:** Metabolic syndrome, Factor analysis, Nutrient patterns

## Abstract

**Background:**

Although there is growing evidence on the association between nutrient patterns and metabolic risk factors, very little is known about the relationship between nutrient patterns and metabolic syndrome (MetS). The aim of this study was to examine the associations of nutrient patterns with MetS among apparently healthy obese adults living in Tabriz, Iran.

**Methods:**

Three hundred and forty-seven apparently healthy obese (BMI ≥ 30 kg/m^2^) adults aged 20–50 years were included in this cross-sectional study. Dietary intake of 38 nutrients was assessed by a validated semi-quantitative food frequency questionnaire (FFQ) of 132 food items. Nutrient patterns were determined using factor analysis. The MetS was defined based on the guidelines of the National Cholesterol Education Program Adult Treatment Panel III (ATP III).

**Results:**

Three major nutrient patterns were extracted: “Mineral based pattern”, “Simple sugar based pattern” and “Fat based pattern”. There was no significant association between nutrient patterns and MetS,

in the crude model even after adjusting for confounders. There was a significant difference between quartiles in the mineral based pattern for free mass (FFM), diastolic blood pressure (DBP), large Waist circumference (WC) and Waist-to-hip ratio (WHR). In the simple sugar based pattern, we observed a significant association for SBP, DBP, and triglyceride (TG) levels. In addition, the fat based pattern was positively associated with BMI, and weight.

**Conclusions:**

We did not observe any significant association of nutrient patterns with the risk of MetS amongst the apparently healthy obese adult's population. Whereas we confirmed the deleterious effect of the simple sugar and fat based patterns on several metabolic risk factors, our findings also showed that the mineral based pattern is related to healthier metabolic factors in an Iranian population. These results should be approved by future studies to recognize any causal relationship between adherence to specific nutrient patterns and MetS.

**Supplementary Information:**

The online version contains supplementary material available at 10.1186/s12902-022-00963-2.

## Background

Metabolic syndrome (MetS) is characterized by the clustering of various common metabolic abnormalities such as impaired glucose tolerance, hyperinsulinism, hypertriglyceridemia, low high density lipoprotein cholesterol (HDL-c), and hypertension [1]. Several studies highlighted the significant relationship between MetS and other conditions such as fatty liver disease, renal disease, cardiovascular disease (CVD), diabetes mellitus, and certain types of cancer, which are known as common complications of obesity [2–5]. Consequently, early prevention and identification of MetS is important to prevent more severe health outcomes.

During the recent years, prevalence of MetS has dramatically increased in several developed and developing countries, likely due to changes in lifestyle and socioeconomic status. Some studies have reported that there was an increasing prevalence of MetS in Asia, particularly in Middle Eastern countries [6, 7], and the increasing trend of MetS also has been observed in Iran, approximately approaching to that of Western nations. A recent systematic review and meta-analysis showed that the prevalence and incidence rate of MetS in Iran was 26% and 97.96%, respectively [8]. One should also consider that although age and genetics are non-modifiable risk factors, lack of physical activity, diet, and excessive energy consumption are potentially modifiable risk factors for MetS [9].

Furthermore, recently researchers have examined the effect of important nutrients on MetS [10, 11]. It has also been reported that increasing dietary zinc, magnesium, potassium, and calcium is related to a lower odds of MetS [12–16]. The majority of epidemiological studies related to MetS and diet have generally focused on single nutrients or food items. Given the complexity of diets and the potential interactions between nutrients, single nutrient approach may be confusing [17, 18]. The overall nutrient patterns may be more important in MetS etiology than assessing the effects of single nutrients due to complexity and synergistic interactions between nutrients. Consequently, the analysis of the nutrient patterns seems to be beneficial in the assessment of the relationship between nutrients and the occurrence of several chronic diseases [19]. Additionally, the benefits of nutrient pattern analysis are that it helps to look at the combination of nutrients in complex biological mechanisms, as well as being easier to use for international studies [20, 21].

Few studies have evaluated the association between nutrient patterns and chronic diseases using factor analysis and provided different results in different populations [22–24]. In a recent published study from Iran, Teymoori et al. [22] has reported that a nutrient pattern rich in dietary potassium, vitamins C and A, B_6_, and fructose was related to a lower risk of hyperinsulinemia, and insulin resistance. Salehi-Abargouei et al. [23] showed that greater adherence to the pattern composed of iron, thiamine, betaine, selenium, niacin, folate, and starch was related to reduced risk of central obesity while another pattern including soluble and insoluble fiber, copper, fructose, glucose, sucrose, and vitamin K and C increased the risk. In fact, nutrient patterns are reflecting the real eating habits of the population and provide more information about probable underlying interactions and synergic effects of nutrients. Therefore, the aim of present study was to assess the associations of major nutrient patterns with the risk of MetS in an apparently healthy obese adult's population in Tabriz-Iran.

## Methods

### Study participants

This cross-sectional study was conducted among 347 apparently healthy obese adults (58.2% males and 41.8% females) in Tabriz, Iran. Eligible participants were enrolled using convenience sampling through announcements that provided general information about inclusion criteria (good health, age 20 to 50 years old, BMI ≥ 30 kg/m^2^) were placed in public places. Exclusion criteria included pregnancy, lactation, menopause, recent surgery such as bariatric, chronic underlying diseases such as CVD, cancer, type 2 diabetes mellitus, hepatic and renal diseases and taking any medication which was effective on weight loss (loop diuretics, corticosteroids or antidepressants drugs).

### Ethics, consent, and permissions

The proposal of this study was approved by the Ethics Committee of Tabriz University of Medical Sciences, Tabriz, Iran (Registration number: IR.TBZME-D.REC.1400.454). Full-informed written consent was taken from all of the participants before participating in the study. This cross-sectional study was prepared in accordance with the STROBE statement.

### General characteristics and anthropometric measurements

Subjects were interviewed on socio-demographic information such as sex, age, smoking, education level, marital status, occupation, medical histories, and family size. All anthropometric measurements were conducted by a well-trained dietitian. Body composition measurements were determined by using bioelectrical impedance analysis (BIA) technology (Tanita, BC-418 MA, Tokyo, Japan). Participant's height was measured to the nearest 0.5 cm using a wall-mounted stadiometer. Physical activity level was assessed using a self-administered short form of the International Physical Activity Questionnaire (IPAQ) [25]. Body weight was measured to the nearest 0.1 kg using a Seca scale (Seca co., Hamburg, Germany), while the subjects were minimally clothed, without shoes or socks. Body mass index (BMI) was calculated as weight divided by height squared (kg/m^2^). Waist circumference (WC) was measured by trained nutritionists at the midpoint between the lower costal margin and the iliac crest using a tape measure to the nearest 0.1 cm. Hip circumference (HC) was also measured over the widest part of the buttocks and was recorded to the nearest 0.1 cm. Waist-to-hip ratio (WHR) was calculated as waist measurement divided by hip measurement. Blood pressure was measured with a standard mercury sphygmo-manometer twice in the same arm after at least 15 min of rest and then mean of the two measurements was used for analysis.

### Definition of the metabolic syndrome (MetS)

MetS was diagnosed if the participants had more than three risk determinants according to the NCEPATP III criteria [26]: TG ≥ 150 mg/dl, FBG ≥ 100 mg/dl, WC ≥ 102 cm in men and ≥ 88 in women, HDL-C < 40 mg/dl in men and < 50 mg/dl in women, and systolic blood pressure (SBP) ≥ 130 mmHg or diastolic blood pressure (DBP) ≥ 85 mmHg.

### Dietary assessments

Trained and experienced dieticians collected dietary data using a validated semi-quantitative food frequency questionnaire (FFQ) of 132 food items, which has been adapted for use in the Iranian population [27]. Participants were asked to report frequency and amount of each food item consumed on a daily, weekly, monthly or yearly basis. Then, the reported frequency of consumed foods and portion sizes for each food item were converted to gram using household measures. Daily energy and nutrient contents were analyzed using the US Department of Agriculture’s (USDA) national nutrient databank. Nevertheless, some local food items that were not available in USDA FCT, was analyzed using The Iranian FCT.

### Biochemical assessment

After an overnight fast, 10 ml venous blood samples were collected from each subject in this study. These whole blood samples were centrifuged at 4500 rpm for 10 min to separate serum and plasma and then immediately aliquoted and frozen at -70 C° until the time of analysis. Measurements of serum total cholesterol (TC), triglyceride (TG), high-density lipoprotein cholesterol (HDL-C), and fasting blood glucose (FBG) were evaluated using a commercial kit (Pars Azmoon, Tehran, Iran). Furthermore, low-density lipoprotein cholesterol (LDL-C) level was estimated by the Friedewald equation [26]. Enzyme-linked immunosorbent assay kits were used to measure serum insulin, plasma AgRP (Sensitivity 1.03 pg/ml), and α-MSH (Sensitivity 5.07 ng/L) concentrations (Bioassay Technology Laboratory, Shanghai Korean Biotech, Shanghai City, China). Homeostatic model assessment for insulin resistance (HOMA-IR) was calculated using the formula: fasting insulin (μ IU/ml) × fasting glucose (mmol/l) /22.5 and quantitative insulin sensitivity check index QUICKI as 1/ [log fasting insulin (μU/mL) + log glucose (mmol/L)].

### Statistical analyses

Statistical analysis of the data was performed using Statistical Package for Social Sciences (version 21.0; SPSS Inc, Chicago IL) at a statistical significance level of < 0.05. The principal component analysis (PCA) with varimax rotation was used to identify nutrient patterns based on the 38 predefined nutrients including carbohydrate, total fat, saturated fatty acids (SFA), polyunsaturated fatty acids (PUFA), monounsaturated fatty acids (MUFA), cholesterol, sugar, fructose, lactose, sucrose, glucose, animal-based protein, plant-based protein, soluble and insoluble fiber, vitamins D, K, A, E, B_12_, C, B_2_, pantothenic acid, thiamine, niacin, folate, pyridoxine, iron, phosphor, zinc, calcium, manganese, copper, magnesium, sodium, selenium, caffeine, and potassium. Statistical correlation between nutrients and adequacy of the data to the factor analysis was tested by Kaiser–Meyer–Olkin (KMO) (0.80) and Barlett's test (P < 0.001). The number of nutrient patterns was determined by considering scree plot and eigenvalues. Factor scores for all participants for each of the extracted factors were computed by summing up intakes of nutrients weighted by their factor loadings. We identified three nutrient patterns based on the scree plot (eigenvalue > 2) and categorized them into quartiles cut off points. The first quartile (Q1) of each nutrient pattern revealed the lower adherence to that certain nutrient pattern. The derived nutrient patterns were labeled based on the nutrients that had high positive loading and also considering the previous studies. Results are presented as frequency (%) for categorical variables and mean ± standard deviation (SD) for continuous variables. The differences in continuous variables across different quartiles of nutrient pattern scores were compared using one-way ANOVA, and the Chi-square test was used to compare the categorical data. To find the relationship between nutrient patterns and odds of MetS, multinational logistic regression was used in different models. Model 1 was adjusted for age, sex, occupation, marital status, education, smoking status, and physical activity and Model 2 was adjusted for Model 1 + energy intake. The first quartile was used as a reference for calculating the odds ratios (OR).

## Results

**Fig. 1:**
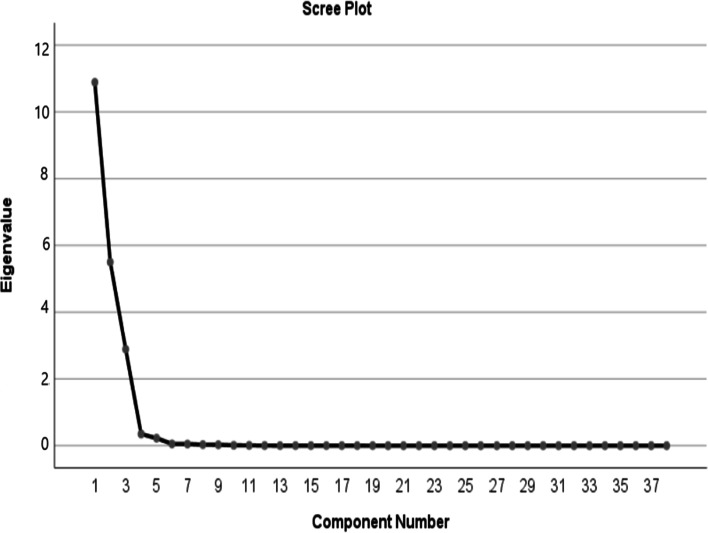
Scree plot of 38 nutrients groups according to its eigenvalues

The mean age of the participants was 40.78 ± 9.23 years, 58.22% of the population was male, about 85.30% of them were married and the mean BMI was 32.62 ± 4.80 kg/m^2^. The prevalence of MetS, hyperglycemia, hypertriglyceridemia, high SBP and DBP, hypo HDL-C, and high WC were 40.82%, 77.51%, 78.07%, 89.42%, 79.82%, 46.52%, and 93.62% respectively. The factor loading matrix of 38 nutrient consumptions and variances of each of three nutrient patterns were presented in Table 1. Using a factor analysis technique, three major nutrient patterns were identified (Fig. 1): (1) Mineral based pattern (variance 27.48%) included vitamins B_1_, B_3_, E, and folate, manganese, selenium, iron, soluble and insoluble fiber, copper, carbohydrate, magnesium, sodium, animal-based protein, plant-based protein, PUFA, and zinc; (2) Simple sugar based pattern (variance 22.43%) included glucose, lactose, fructose, sugar, sucrose, phosphorus vitamins C, B_5_, and B_6_; and (3) Fat based pattern (variance 16.94%) included saturated fatty acid, fat, MUAF, potassium, calcium, cholesterol, vitamins A, B_2_, D, K, B_12_, and caffeine. In combination the nutrient patterns explained 66.85% of the whole variance in nutrient intakes (Table 1). The baseline characteristics of the participants across quartiles of nutrient patterns are shown in Table 2. There were significant differences in fat free mass (FFM) (*P * =  0.02), protein, fat, and carbohydrate intake (*P * =  0.01) across different quartiles of mineral based pattern. Those in the top quartile of the simple sugar based pattern were older (43.23 ± 10.49 versus 38.32 ± 8.89 y, *P*  <  0.04) and were more likely to have higher intake of protein, fat, and carbohydrate (*P* =  0.01). The distribution of education was significantly different among quartiles of the simple sugar based pattern (*P * =  0.01); those who had a higher level of education were assigned to the top quartile of this pattern. Across different quartiles of fat based pattern, there were significant differences in age, BMI, weight, and protein, fat, and carbohydrate intake (*P * =  0.01). Table 3 presents the multivariate adjusted means for metabolic variables according to quartiles of factor scores of major nutrient patterns. We found that participants in the higher quartiles of the mineral based pattern had significantly lower DBP (*P * =  0.04), WC (*P * =  0.05), and WHR (*P * =  0.04) compared to first quartiles in the crude model even after adjusting for confounders. Higher adherence to the single sugar based pattern was associated with higher SBP, DBP, and TG (*P * <  0.05). After adjusting for confounders significant association remained for DBP (*p * =  0.01). A significant lower SBP, DBP, and QUICKI level was seen in the upper quartile of the fat based pattern compared with that in the lowest quartile. After adjusting for confounders significant association remained for SBP (*p* = 0.01).

**Table 1:** Factor loading matrix for the nutrients representing the three major nutrient patterns in a cross-sectional study of MetS in Iran (*N* = 347)

Fat based	Simple sugar	Mineral based	Nutrients
_	0.30	0.86	Vitamin B_1_
_	_	0.82	Manganese
_	_	0.81	Selenium
-	0.31	0.81	Folate
_	0.36	0.80	Vitamin B_3_
_	0.38	0.78	Iron
_	-	0.75	Soluble fiber
_	0.32	0.73	Insoluble fiber
0.36	0.33	0.71	Copper
_	0.62	0.69	Carbohydrate
0.35	0.49	0.65	Magnesium
_	_	0.63	Sodium
0.45	0.46	0.62	Animal-based protein
0.43	0.32	0.60	Plant-based protein
0.51	_	0.59	PUFA
0.49	0.36	0.56	Zinc
0.36	_	0.48	Vitamin E
_	0.86	_	Glucose
_	0.85	_	Vitamin C
_	0.84	_	Fructose
0.12	0.80	_	Sugar
0.43	0.74	0.36	Vitamin B_5_
_	0.69	0.51	Vitamin B_6_
0.60	0.51	0.37	Calcium
0.47	0.57	0.54	Phosphorus
0.42	0.56	_	Lactose
_	0.46	_	Sucrose
0.79	0.34	0.35	Potassium
0.75	_	_	Saturated fatty acid
0.70	0.25	0.52	Fat
0.66	_	0.49	MUFA
0.65	_	_	Vitamin B_12_
0.62	_	_	Cholesterol
0.60	_	_	Vitamin A
0.60	0.46	0.51	Vitamin B_2_
0.57	_	_	Vitamin D
0.32	_	_	Vitamin K
0.31	_	0.30	Caffeine
16.94	22.43	27.48	Explained variance (%)
66.85	49.91	27.48	Cumulative explained variance (%)

**Table 2:** The baseline characteristics of study population across quartiles of nutrient patterns in a cross-sectional study of MetS in Iran (*n* = 347)

Fat based					Simple sugar based					Mineral based						Variable
*p*	Q4 (*n*=87)	Q3 (*n*=87)	Q2 (*n*=86)	Q1 (*n*=87)	*p*	Q4 (*n*=87)	Q3 (*n*=87)	Q2 (*n*=86)	Q1 (*n*=87)	*p*	Q4 (*n*=87)	Q3 (*n*=87)	Q2 (*n*=86)	Q1 (*n*=87)		
0.01	38±9	39±8	41±9	43±9	0.04	43±10	41±8	39±8	38±8	0.44	40±9	39±8	41±10	41±8		Age (years)
0.56	55(62.4)	52(58.3)	47(51.8)	50(56.0)	0.57	48(54.1)	52(59.0)	55(62.4)	47(52.9)	0.09	55(62.4)	58(65.1)	46(52.9)	43(48.2)		Gender (male )
0.72	33.8±10.4	32.9±8.2	33.6±9.1	35.2±8.1	0.11	36.0±8.3	35.0±10.5	31.4±8.4	34.0±8.7	0.27	33.7±9.7	32.3±8.5	33.2±7.9	35.9±9.7		FM (kg)
0.77	63.3±12.6	62.3±12.6	61.8±12.4	60.6±11.9	0.28	62.9±11.8	63.8±11.6	63.8±12.9	59.8±12.3	0.02	59.0±13.3	65.8±11.8	59.7±11.2	63.2±11.3		FFM (kg)
0.08					0.66					0.18						Physical activity (%)
	43(50.0)	31(35.2)	38(43.6)	60(68.6)		43(50.0)	43(50.0)	34(40.0)	45(52.3)		39(45.8)	41(46.9)	55(63.9)	34(38.6)		Low
	20(23.3)	32(37.0)	24(28.2)	17(20.0)		22(25.0)	18(20.5)	29(32.7)	26(29.2)		24(27.1)	19(22.4)	24(27.8)	30(34.1)		Medium
	24(26.7)	24(27.8)	24(28.2)	10(11.4)		22(25.0)	26(29.5)	23(27.3)	16(18.5)		24(27.1)	27(30.6)	7(8.3)	23(27.3)		High
0.45					0.27					0.31						Marital status (%)
	69(78.8)	78(89.3)	75(87.1)	76(86.9)		72(82.4)	75(86.7)	78(90.6)	72(82.4)		75(85.9)	69(79.5)	74(85.9)	78(90.6)		Married
0.44					0.01					0.92						Education (%)
	1(1.1)	0(0)	2(2.6)	0(0)		0(0)	0(0)	0(0)	3(3.1)		9(10.4)	4(4.1)	8(9.3)	7(8.3)		Illiterate
	21(25.4)	15(16.7)	24(28.2)	17(20.0)		11(12.5)	16(18.7)	18(20.0)	27(30.8)		50(56.9)	46(53.1)	39(45.4)	40(45.4)		≤ High school/diploma
	66(76.4)	72(83.3)	60(69.3)	70(80.0)		76(87.5)	71(81.3)	68(80.0)	57(66.1)		28(32.7)	37(42.8)	39(45.3)	41(46.3)		≥ College degree
0.01	130±44	101±26	84±24	83±29	0.01	125±44	104±31	88±25	81±26	0.01	129±42	100±27	93±27	76±26		Protein (g)
0.01	522±184	433±128	403±151	448±187	0.01	613±179	459±130	384±98	351±126	0.01	609±175	463±132	402±110	332±116		Carbohydrate (g)
0.01	150±52	101±26	80±25	69±28	0.01	112±52	103±46	86±34	99±87	0.01	131±51	106±41	84±35	79±39		Fat (g)

**Table 3:** The multivariate adjusted means for the Metabolic variables according to quartiles of major nutrient patterns in in a cross-sectional study of MetS in Iran (*n* = 347)

Fat based						Simple sugar based							Mineral based							Variables	
P2	*P1*	Q4 (*n*=87)	Q3 (*n*=87)	Q2 (*n*=86)	Q1 (*n*=87)		*P2*	*P1*	Q4 (*n*=87)	Q3 (*n*=87)	Q2 (*n*=86)	Q1 (*n*=87)		*P2*	*P1*	Q4 (*n*=87)	Q3 (*n*=87)	Q2(*n*=86)	Q1 (*n*=87)		
0.85	0.93	33(37.6)	34(39.3)	37(42.4)	35(39.8)		0.22	0.35	41(47.1)	36(41.0)	31(35.7)	31(35.3)		0.83	0.92	33(37.6)	34(38.6)	35(40.5)	37(42.4)	MetS n(%)	
0.09	0.11	108.7±10.6	106.8±9.4	105.7±9.1	105.4±8.8		0.71	0.77	105.7±9.3	106.9±8.2	107.4±8.0	106.7±11.3		0.03	0.05	105.2±8.5	105.3±10.2	107.9±9.8	108.3±9.4	WC (cm)	
0.04	0.01	33.7±4.9	33.5±4.3	31.5±5.0	31.8±4.5		0.12	0.14	31.8±5.4	33.1±4.8	32.4±3.8	33.3±4.8		0.06	0.08	33.9±4.9	32.4±5.7	31.5±4.2	32.9±5.1	BMI(kg/m^2^)	
0.03	0.01	95.7±15.2	95.0±15.2	88.1±14.6	89.3±13.8		0.30	0.31	89.7±16.6	93.7±13.4	92.9±12.6	92.0±14.5		0.18	0.24	93.4±13.3	93.8±14.4	89.8±13.6	91.3±16.2	Weight (kg)	
0.11	0.12	0.94±0.07	0.92±0.09	0.93±0.06	0.91±0.07		0.86	0.89	0.93±0.06	0.93±0.07	0.93±0.08	0.92±0.07		0.02	0.04	0.91±0.09	0.93±0.06	0.92±0.07	0.95±0.07	WHR (m)	
0.12	0.13	95.5±28.7	94.9±19.2	89.7±11.7	90.8±12.3		0.62	0.65	92.3±27.8	92.8±17.8	91.6±16.2	90.3±12.5		0.84	0.96	92.1±18.2	93.0±18.2	92.4±14.5	93.5±26.5	FBG (mg/dl)	
0.47	0.49	14.1±7.9	17.0±8.2	17.6±14.8	16.3±21.4		0.55	0.56	18.4±20.5	15.1±8.3	15.8±13.1	15.6±11.1		0.28	0.32	17.6±19.7	14.1±7.7	14.7±7.7	17.7±13.5	Insulin (U/mL)	
0.79	0.81	3.4±2.6	3.5±2.0	3.9±3.4	3.7±4.7		0.51	0.53	4.4±4.5	3.5±2.0	3.9±3.9	3.5±2.3		0.21	0.32	3.9±4.2	3.3±1.9	3.3±1.8	4.2±3.9	HOMA-IR	
0.56	0.04	0.31±0.02	0.32±0.03	0.33±0.03	0.33±0.04		0.20	0.21	0.33±0.04	0.32±0.02	0.32±0.02	0.33±0.03		0.10	0.11	0.32±0.03	0.32±0.02	0.32±0.02	0.32±0.04	QUICKI	
0.01	0.01	119±18	121±13	126±16	123±14		0.45	0.04	128±14	123±19	121±15	118±14		0.14	0.18	120±20	121±14	125±14	122±13	SBP (mmHg)	
0.24	0.01	78±13	79±11	85±10	82±9		0.01	0.01	85±9	81±13	81±11	78±11		0.03	0.04	79±13	80±10	84±11	82±10	DBP (mmHg)	
0.61	0.63	145±98	147±90	148±76	162±106		0.34	0.03	171±104	157±95	132±68	141±85		0.22	0.20	143±77	153±105	166±79	138±72	TG (mg/dl)	
0.46	0.48	187±35	193±33	190±40	195±38		0.85	0.87	194±40	190±37	190±35	191±33		0.17	0.25	187±32	194±36	196±35	188±41	TC (mg/dl)	
0.35	0.37	118±31	124±31	124±32	127±33		0.32	0.34	128±34	121±32	123±28	120±32		0.08	0.09	117±30	123±30	129±33	123±32		LDL-C (mg/dl)
0.12	0.16	44±10	44±9	43±10	43±10		0.36	0.38	43±9	42±8	44±10	44±8		0.17	0.18	43±8	44±10	41±8	45±10		HDL-C (mg/dl)

However, there were no significant differences in prevalence of MetS across different quartiles of nutrient patterns (*P*  >  0.05). Odds of MetS with different nutrient patterns in two adjusted and crude models are presented in odds ratios (OR) and 95% confidence intervals (CI) (Table 4). Logistic regression analysis showed that no significant association was found between MetS and the nutrient patterns in both crude and adjusted models.

**Table 4:** Odd’s ratio (OR) and confidence interval (CI) for MetS and its components according to quartiles (Q) of nutrient patterns in a cross-sectional study of MetS in Iran (*n* = 347)

Fat based					Simple sugar based					Mineral based					Variable
*p*	Q4 (*n*=87)	Q3 (*n*=87)	Q2 (*n*=87)	Q1 (*n*=86)	*p*	Q4 (*n*=87)	Q3 (*n*=87)	Q2 (*n*=86)	Q1 (*n*=87)	*p*	Q4 (*n*=87)	Q3 (*n*=87)	Q2 (*n*=86)	Q1 (*n*=87)	
															MetS^a^
0.24	1.28(0.82-1.65)	1.16(075-1.79)	1.01 (0.65, 1.56)	1 (Ref.)	0.74	0.79 (0.52-1.18)	0.86 (0.57-1.29)	0.89 (0.59-1.33)	1 (Ref.)	0.88	0.97 (0.65-1.46)	0.93 (0.62-1.40)	1.10 (0.73-1.64)	1 (Ref.)	Crude
0.12	0.62(0.28-0.91)	0.56(0.32-0.99)	0.68 (0.40, 1.14)	1 (Ref.)	0.06	0.84 (0.54-1.30)	0.88 (0.57-1.36)	0.79 (0.52-1.18)	1 (Ref.)	0.18	1.39(0.85-2.27)	0.98(0.59-1.59)	1.09(0.67-1.76)	1 (Ref.)	Model 1^b^
0.36	0.60(0.29-0.84)	0.53(0.28-0.75)	0.59 (0.43, 1.22)	1 (Ref.)	0.62	0.77 (0.49-1.20)	0.78 (0.49-1.23)	0.78 (0.50-1.22)	1 (Ref.)	0.73	1.02 (0.65-1.58)	0.87 (0.56-1.35)	1.12(0.72-1.72)	1 (Ref.)	Model 1^c^

## Discussions

In the present cross-sectional study, according to the NCEPATP III criteria [26], we reported that prevalence of MetS was 40.82% in a randomly-selected sample of the apparently healthy obese adult's population of north-west of Iran, which is similar with that reported in a previous study [28].

To assess the relationship with MetS**,** we first extracted three major nutrient patterns which described the dietary habits of the study participants. The composition of these nutrient patterns partly overlapped with those examined by two previous cross-sectional and cohort studies in Iran. Salehi-Abargouei et al. [23] found three major nutrient patterns in a sample of Iranian adults. In another study by Teymoori et al. [22] five major nutrient patterns were also found which were different from patterns obtained in our study. However, it should be keep in mind that since nutrient patterns are extracted from data obtained in the studied population with different dietary habits, consequently it is not expected that the nutrient patterns would be completely in accordance with each other.

We found that the recognized major nutrient patterns were not associated with MetS, but there were some associations found with MetS components. As the whole, the interactions between nutrients with MetS protecting, inducing, and neutral effects resulted in a non-significant relationship between nutrient patterns and MetS in our study.

The first pattern-named “Mineral based pattern”-was characterized by high intake of manganese, selenium, iron, copper, magnesium, sodium, zinc, soluble and insoluble fiber, carbohydrate, protein (animal and plant -based), PUFA, vitamins E, B_1_, B_3_, and folate. In the current study we observed that FFM, DBP, WC, and WHR decreased with increasing adherence to mineral based pattern among obese adults living in Tabriz, Iran. Our findings are in line with previous reports indicating the protective effect of selenium, copper, manganese and vitamin E against the risk of hypertension, obesity, and diabetes [13, 29–32]. Since the components of the mineral based pattern are abundantly contained in fruits and vegetables, the current findings are in agreement with reports that the healthy dietary pattern, characterized by high consumption of fruits, vegetables, cereals, and legumes, was negatively associated with MetS components in cohort [33] and cross-sectional studies [16, 34, 35]. For instance, studies in USA and China reported that diets with a high intake of whole grains, vegetables, and fruits were associated with a lower BMI, WC and blood pressure [36, 37]. Freisling et al. [38] found that nutrients from plant sources such as vitamin C, folate, fiber, and beta-carotene are associated with lower weight, however, vitamin B_2_, calcium, phosphorus, and protein are associated with obesity in adults. The concept that MetS is an inflammatory state may explain the role of vitamin E, PUFA, selenium, and magnesium. In a study done by Mah et al. [38], it was observed that MetS patients had impaired absorption of dietary vitamin E compared to healthy adults. Moreover, in an animal study, vitamin E administration effectively reduced hypertriglyceridemia and insulin resistance, which was attributed to the down-regulation of inflammatory response and signaling pathways [32]. Like our study, Barzegar-Amini et al. [39], in a randomized trial of 363 patients with dyslipidemia, have found that serum vitamin E is inversely related to WC; but the change observed in weight was not significant. In study by Wallstrom et al. [40] serum α-tocopherol is positively associated with WC and WHR after adjustment for body fat. Several studies showed that omega-3 and omega-6 fatty acids intake were inversely associated with MetS [41, 42]. In a systematic review by Tortosa-Caparros and colleagues [43], total PUFA intake were inversely related to hypertension and positively related to abdominal obesity.

Several studies have found the association of single nutrients such as, sodium, calcium, potassium, and magnesium with hypertension [44–46]. It has been demonstrated that sodium intake is strictly associated with raised blood pressure levels. As described in the study by Grillo et al. [45] salt intake exerts hypertensive effects by various mechanisms such as, water retention, increase in systemic peripheral resistance, endothelial dysfunction, and sympathetic hyperactivity. Also, it has been shown that a shift from low-to high- calcium intake reduced the blood pressure by regulation of renin-angiotensin system and parathyroid hormone signaling pathway [46]. Nevertheless, when the relationship of whole nutrients with MetS components were assessed, the interaction of potentially harmful and useful nutrients is unavoidable and their effects may be neutralized by each other.

Another major nutrient pattern newly recognized in our study is simple sugar based pattern and consisted of high intake of glucose, lactose, fructose, sucrose, sugar, phosphorus, vitamins C, B_5_, and B_6_. As expected, our results reported increasing SBP, DBP, and TG with increasing adherence to simple sugar based pattern in our study. The direct link of dietary fructose, consumed from high-fructose beverages such as fruit juices, and soft drinks, with TG levels is well documented [47]. These results are in accordance with previous reports; Khayyatzadeh et al. [24] found three major nutrient patterns in a sample of Iranian adults. They reported that a nutrient pattern characterized by simple sugars, increased TG levels in women; nevertheless, patterns with high selenium and vitamins A and B decreased TG levels in men. Two systematic review and meta-analysis of clinical trials in 2014 and 2015 concluded that fructose in an isocaloric diet does not increase TG levels compared to other carbohydrates; nevertheless, if the calorie consumption increases, fructose increases the synthesis of TG [48, 49]. In human and animal studies, fructose has been found to increase blood pressure [50–52]. It has been suggested that high sugar and soft drinks can lead to hypertension by inactivation of nitric oxide (NO), down-regulating the level of NO synthase protein, and sympathetic hyperactivity [53, 54]. In another study by Chikowore et al. [55] in South Africa three nutrient patterns were also obtained. The pattern including high consumption of plant proteins, vitamin B_1_, and zinc and a pattern which was rich in fiber, vitamin B group, and carbohydrates were related to lower serum hemoglobin A1c (HbA1c), and FBG, in rural men and women, respectively. Differences in study design, patient population studied, races, and study size could contribute to mixed results. Several reports revealed that vitamin B group and its synergistic effect play an important role in fat and lipid metabolism. This pattern has a higher amount of vitamin B5, vitamin B6 as well as overloaded simple sugars. These components are closely associated with the synthesis of fat from protein and carbohydrates [56]. Generally, a nutrient pattern with high vitamins and simple sugar, specifically vitamin B group which shows a synergist effect with other nutrients can be a main factor leading to high SBP, DBP, and TG.

The third pattern-named “Fat based pattern”-was characterized by high intake of saturated fatty acid, fat, MUAF, potassium, calcium, cholesterol, caffeine, vitamins A, B_2_, D, K, and B12. The combination of nutrients with neutral (vitamins K and A), MetS-protecting (potassium, calcium and vitamins B12, B2, and D) and MetS-inducing (SFA, and dietary cholesterol) effects in this pattern made our interpretation more difficult. It should be noted that participants with MetS may change their diet and lifestyle to alleviate MetS components, and this issue may be a reason for the existence of MetS protecting nutrients in fat based pattern.

In this study, fat based nutrient pattern was associated with higher BMI, and weight. It is well established that high fat diet has high energy density; therefore it may contribute to excessive caloric intake and obesity as risk factors from lifestyle-associated diseases in humans. Dietary intakes of saturated fatty acid and cholesterol have been positively associated with MetS in the prior studies [57, 58]. However, there is considerable epidemiological evidence showing an inverse relationship between vitamins D, B2, B12, potassium, and calcium with MetS [59–61]. We also detected a significant decrease in SBP across quartiles of fat based pattern in adjusted model; this can explained by high factor loadings for some beneficial nutrients, such as vitamin D, calcium, and MUAF.

The fat based pattern had no significant association with serum lipids and glucose which may be due to interactions between nutrient components. For example, in this pattern, calcium or dairy products can reduce the intestinal absorption of fat by the formation of insoluble soap; these nutrients appear to counteract the effects of each other [62]. However, nutrients of the fat based pattern, such as saturated fatty acid, fat, and cholesterol, have enhancing effects on some aspects of lipid and carbohydrate metabolism in the human body. In previous study by Iwasaki et al. [63] a fats and fat-soluble vitamins nutrient pattern characterized by omega-3, omega-6 fatty acids, vitamin E, and MUFA was associated with increased risk of MetS, obesity, and high blood pressure. A number of studies have shown that Western dietary pattern including higher intake of red/organ meat, processed meat, canned fruits, refined grains, and high-fat cheese is significantly associated with increased risk of MetS [17, 34, 64]. Previous studies showed that nutrient patterns with high amount of carbohydrate and fat are associated with a higher risk of obesity and excess body weight [65]. Cai et al. [61], in a meta-analysis of eight studies, evaluated the relations between potassium and obesity/MetS. Their results indicated that high dietary potassium consumption was related to lower risk of MetS (OR = 0.75; 95% CI: 0.50–0.97). In addition, it is well noted that increased potassium intake decreases hypertension by counteracting the effects of sodium [66]. Unlike our prediction to find the relationship between fat based pattern and MetS, no association was observed. This result can be attributed to a higher amount of MUFA, potassium, calcium, vitamin D, and caffeine which are well-known as protective nutrients against MetS.

The present study was the first comprehensive study with a large sample size to assess the relationship between nutrient patterns and MetS in apparently healthy obese adults in Iran. On the other hand, the current study also has some inevitable limitations. First of all, since this was a cross-sectional study, causal relations cannot be made. However, additional studies with prospective designs are necessary to confirm our results. Second, although this study used a validated FFQ, measurement error and recall bias in the assessment of dietary intake may have been unavoidable. Finally, owing to the unique nature of the derived nutrient patterns in the target population, the findings may not be generalizable to other populations with different ethnic backgrounds.

## Conclusions

In conclusion, based on the results of the current study, among the general population of north-west of Iran, three main nutrient patterns including mineral based, simple sugar based, and fat based nutrient patterns were found. We did not observe any significant association of nutrient patterns with the risk of MetS amongst the apparently healthy obese adult's population. Whereas we confirmed the deleterious effect of the simple sugar and fat based patterns on several metabolic risk factors, our findings also showed that the mineral based pattern is related to healthier metabolic factors in an Iranian population. Future longitudinal studies are needed to evaluate the key players in MetS development.

## Supplementary Information

**Additional file 1. **

**Additional file 2. **

## Data Availability

All of the data are available with reasonable request from the corresponding author.

## Abbreviations

BMI: Body mass index
CI: Confidence interval
DBP: Diastolic blood pressure
FBG: Fasting blood glucose
FFQ: Food frequency questionnaire
HDL-C: High-density lipoprotein cholesterol
IPAQ: International physical activity questionnaire
MetS: Metabolic syndrome; PA: Physical activity
PCA: Principal Components Analysis
SBP: Systolic blood pressure
SD: Standard Deviations
TC: Total cholesterol
TG: Triglyceride
WC: Waist circumference
WHR: Waist hip ratio
