# Supplementary material for: Nutrient pattern analysis of mineral based, simple sugar based, and fat based diets and risk of metabolic syndrome: a comparative nutrient panel
Source: BMC Endocr Disord. 2022 Mar 2;22:51. doi: 10.1186/s12902-022-00963-2 (PMC8889682; doi:10.1186/s12902-022-00963-2)
Supplement: Supplementary file 1 — Additional file 1. [file 12902_2022_963_MOESM1_ESM.docx]

Table 1 Factor loading matrix for the nutrients representing the three major nutrient patterns in a cross-sectional study of MetS in Iran (N=347)

| Fat based | Simple sugar | Mineral based | Nutrients |
| --- | --- | --- | --- |
| _ | 0.30 | **0.86** | Vitamin B_1_ |
| _ | **_** | **0.82** | Manganese |
| _ | **_** | **0.81** | Selenium |
| - | 0.31 | **0.81** | Folate |
| _ | 0.36 | **0.80** | Vitamin B_3_ |
| _ | 0.38 | **0.78** | Iron |
| _ | **-** | **0.75** | Soluble fiber |
| _ | 0.32 | **0.73** | Insoluble fiber |
| 0.36 | 0.33 | **0.71** | Copper |
| _ | 0.62 | **0.69** | Carbohydrate |
| 0.35 | 0.49 | **0.65** | Magnesium |
| _ | **_** | **0.63** | Sodium |
| 0.45 | 0.46 | **0.62** | Animal-based protein |
| 0.43 | 0.32 | **0.60** | Plant-based protein |
| 0.51 | **_** | **0.59** | PUFA |
| 0.49 | 0.36 | **0.56** | Zinc |
| 0.36 | **_** | **0.48** | Vitamin E |
| _ | **0.86** | **_** | Glucose |
| _ | **0.85** | _ | Vitamin C |
| _ | **0.84** | **_** | Fructose |
| 0.12 | **0.80** | **_** | Sugar |
| 0.43 | **0.74** | 0.36 | Vitamin B_5_ |
| _ | **0.69** | 0.51 | Vitamin B_6_ |
| 0.60 | **0.51** | 0.37 | Calcium |
| 0.47 | **0.57** | 0.54 | Phosphorus |
| 0.42 | **0.56** | _ | Lactose |
| _ | **0.46** | **_** | Sucrose |
| 0.79 | 0.34 | 0.35 | Potassium |
| 0.75 | **_** | _ | Saturated fatty acid |
| 0.70 | 0.25 | 0.52 | Fat |
| 0.66 | _ | 0.49 | MUFA |
| 0.65 | **_** | **_** | Vitamin B_12_ |
| 0.62 | _ | _ | Cholesterol |
| 0.60 | **_** | **_** | Vitamin A |
| 0.60 | 0.46 | 0.51 | Vitamin B_2_ |
| 0.57 | **_** | _ | Vitamin D |
| 0.32 | **_** | **_** | Vitamin K |
| 0.31 | _ | 0.30 | Caffeine |
| 16.94 | 22.43 | 27.48 | **Explained variance (%)** |
| 66.85 | 49.91 | 27.48 | **Cumulative explained variance (%)** |

Loading values of 0.10 or greater are indicated by Bold values. MetS, Metabolic syndrome; PUFA, Polyunsaturated fatty acid; MUFA, Monounsaturated fatty acid.
