# Supplementary material for: Nutrient pattern analysis of mineral based, simple sugar based, and fat based diets and risk of metabolic syndrome: a comparative nutrient panel
Source: BMC Endocr Disord. 2022 Mar 2;22:51. doi: 10.1186/s12902-022-00963-2 (PMC8889682; doi:10.1186/s12902-022-00963-2)
Supplement: Supplementary file 2 — Additional file 2. [file 12902_2022_963_MOESM2_ESM.docx]

**Table 2** The baseline characteristics of study population across quartiles of nutrient patterns in a cross-sectional study of MetS in Iran (n=347)

| Mineral based  **p** Simple sugar based **p** Fat based **p** | | | | | | | | | | | | | | | | | | **Variable** |
| --- | --- | --- | --- | --- | --- | --- | --- | --- | --- | --- | --- | --- | --- | --- | --- | --- | --- | --- |
| **Q1** (n=87) **Q2** (n=86) **Q3** (n=87) **Q4** (n=87) **Q1** (n=87) **Q2** (n=86) **Q3** (n=87) **Q4** (n=87) **Q1** (n=87) **Q2** (n=86) **Q3** (n=87) **Q4** (n=87) | | | | | | | | | | | | | | | | | |  |
| **0.01** | 38±9 | 39±8 | 41±9 | 43±9 | **0.04** | 43±10 | 41±8 | 39±8 | 38±8 | 0.44 | 40±9 | | 39±8 | | 41±10 | | 41±8 | Age (years) |
| 0.56 | 55(62.4) | 52(58.3) | 47(51.8) | 50(56.0) | 0.57 | 48(54.1) | 52(59.0) | 55(62.4) | 47(52.9) | 0.09 | 55(62.4) | | 58(65.1) | | 46(52.9) | | 43(48.2) | Gender (male ) |
| 0.72 | 33.8±10.4 | 32.9±8.2 | 33.6±9.1 | 35.2±8.1 | 0.11 | 36.0±8.3 | 35.0±10.5 | 31.4±8.4 | 34.0±8.7 | 0.27 | 33.7±9.7 | | 32.3±8.5 | | 33.2±7.9 | | 35.9±9.7 | FM (kg) |
| 0.77 | 63.3±12.6 | 62.3±12.6 | 61.8±12.4 | 60.6±11.9 | 0.28 | 62.9±11.8 | 63.8±11.6 | 63.8±12.9 | 59.8±12.3 | **0.02** | 59.0±13.3 | | 65.8±11.8 | | 59.7±11.2 | | 63.2±11.3 | FFM (kg) |
| 0.08 |  |  |  |  | 0.66 |  |  |  |  | 0.18 | **Physical activity (%)** | | | | | | | |
|  | 43(50.0) | 31(35.2) | 38(43.6) | 60(68.6) |  | 43(50.0) | 43(50.0) | 34(40.0) | 45(52.3) |  | 39(45.8) | 41(46.9) | | 55(63.9) | | 34(38.6) | | Low |
|  | 20(23.3) | 32(37.0) | 24(28.2) | 17(20.0) |  | 22(25.0) | 18(20.5) | 29(32.7) | 26(29.2) |  | 24(27.1) | 19(22.4) | | 24(27.8) | | 30(34.1) | | Medium |
|  | 24(26.7) | 24(27.8) | 24(28.2) | 10(11.4) |  | 22(25.0) | 26(29.5) | 23(27.3) | 16(18.5) |  | 24(27.1) | 27(30.6) | | 7(8.3) | | 23(27.3) | | High |
| 0.45 |  |  |  |  | 0.27 |  |  |  |  | 0.31 |  |  | |  | | **Marital status (%)** | | |
|  | 69(78.8) | 78(89.3) | 75(87.1) | 76(86.9) |  | 72(82.4) | 75(86.7) | 78(90.6) | 72(82.4) |  | 75(85.9) | 69(79.5) | | 74(85.9) | | 78(90.6) | | Married |
| 0.44 |  |  |  |  | **0.01** |  |  |  |  | 0.92 |  |  | |  | |  | | **Education (%)** |
|  | 1(1.1) | 0(0) | 2(2.6) | 0(0) |  | 0(0) | 0(0) | 0(0) | 3(3.1) |  | 9(10.4) | 4(4.1) | | 8(9.3) | | 7(8.3) | | Illiterate |
|  | 21(25.4) | 15(16.7) | 24(28.2) | 17(20.0) |  | 11(12.5) | 16(18.7) | 18(20.0) | 27(30.8) |  | 50(56.9) | 46(53.1) | | 39(45.4) | | ≤ High school/diploma 40(45.4) | | |
|  | 66(76.4) | 72(83.3) | 60(69.3) | 70(80.0) |  | 76(87.5) | 71(81.3) | 68(80.0) | 57(66.1) |  | 28(32.7) | 37(42.8) | | 39(45.3) | | ≥ College degree 41(46.3) | | |
| **0.01** | 130±44 | 101±26 | 84±24 | 83±29 | **0.01** | 125±44 | 104±31 | 88±25 | 81±26 | **0.01** | 129±42 | 100±27 | | 93±27 | | 76±26 | | Protein (g) |
| **0.01** | 522±184 | 433±128 | 403±151 | 448±187 | **0.01** | 613±179 | 459±130 | 384±98 | 351±126 | **0.01** | 609±175 | 463±132 | | 402±110 | | Carbohydrate (g) 332±116 | | |
| **0.01** | 150±52 | 101±26 | 80±25 | 69±28 | **0.01** | 112±52 | 103±46 | 86±34 | 99±87 | **0.01** | 131±51 | 106±41 | | 84±35 | | 79±39 | | Fat (g) |

P-value obtained using one-way ANOVA for continuous variables and Chi-square test for categorical variables. Categorical and continuous variables data are presented as number (percent) and mean (SD). *Abbreviations*: BMI; body mass index, FFM; Fat free mass, FM; Fat mass.

**Table 3** The multivariate adjusted means for the Metabolic variables according to quartiles of major nutrient patterns in in a cross-sectional study of MetS in Iran (n=347)

| **P2** | **P1** |  | | **Mineral based P1 P2 Simple sugar based P1 P2 Fat based** | | | | | | | | | | | | | | | **Variables** |
| --- | --- | --- | --- | --- | --- | --- | --- | --- | --- | --- | --- | --- | --- | --- | --- | --- | --- | --- | --- |
|  |  | **Q4** (n=87) | **Q3** (n=87) | | **Q2** (n=86) | **Q1** (n=87) |  |  | **Q4** (n=87) | **Q3** (n=87) | **Q2** (n=86) | **Q1** (n=87) |  |  | **Q4** (n=87) | **Q3** (n=87) | **Q2**(n=86) | **Q1** (n=87) |  |
| 0.85 | 0.93 | 33(37.6) | 34(39.3) | | 37(42.4) | 35(39.8) | 0.22 | 0.35 | 41(47.1) | 36(41.0) | 31(35.7) | 31(35.3) | 0.83 | 0.92 | 33(37.6) | 34(38.6) | 35(40.5) | 37(42.4) | MetS n(%) |
| 0.09 | 0.11 | 108.7±10.6 | 106.8±9.4 | | 105.7±9.1 | 105.4±8.8 | 0.71 | 0.77 | 105.7±9.3 | 106.9±8.2 | 107.4±8.0 | 106.7±11.3 | **0.03** | **0.05** | 105.2±8.5 | 105.3±10.2 | 107.9±9.8 | 108.3±9.4 | WC (cm) |
| **0.04** | **0.01** | 33.7±4.9 | 33.5±4.3 | | 31.5±5.0 | 31.8±4.5 | 0.12 | 0.14 | 31.8±5.4 | 33.1±4.8 | 32.4±3.8 | 33.3±4.8 | 0.06 | 0.08 | 33.9±4.9 | 32.4±5.7 | 31.5±4.2 | 32.9±5.1 | BMI(kg/m^2^) |
| **0.03** | **0.01** | 95.7±15.2 | 95.0±15.2 | | 88.1±14.6 | 89.3±13.8 | 0.30 | 0.31 | 89.7±16.6 | 93.7±13.4 | 92.9±12.6 | 92.0±14.5 | 0.18 | 0.24 | 93.4±13.3 | 93.8±14.4 | 89.8±13.6 | 91.3±16.2 | Weight (kg) |
| 0.11 | 0.12 | 0.94±0.07 | 0.92±0.09 | | 0.93±0.06 | 0.91±0.07 | 0.86 | 0.89 | 0.93±0.06 | 0.93±0.07 | 0.93±0.08 | 0.92±0.07 | **0.02** | **0.04** | 0.91±0.09 | 0.93±0.06 | 0.92±0.07 | 0.95±0.07 | WHR (m) |
| 0.12 | 0.13 | 95.5±28.7 | 94.9±19.2 | | 89.7±11.7 | 90.8±12.3 | 0.62 | 0.65 | 92.3±27.8 | 92.8±17.8 | 91.6±16.2 | 90.3±12.5 | 0.84 | 0.96 | 92.1±18.2 | 93.0±18.2 | 92.4±14.5 | 93.5±26.5 | FBG (mg/dl) |
| 0.47 | 0.49 | 14.1±7.9 | 17.0±8.2 | | 17.6±14.8 | 16.3±21.4 | 0.55 | 0.56 | 18.4±20.5 | 15.1±8.3 | 15.8±13.1 | 15.6±11.1 | 0.28 | 0.32 | 17.6±19.7 | 14.1±7.7 | 14.7±7.7 | 17.7±13.5 | Insulin **(**U/mL**)** |
| 0.79 | 0.81 | 3.4±2.6 | 3.5±2.0 | | 3.9±3.4 | 3.7±4.7 | 0.51 | 0.53 | 4.4±4.5 | 3.5±2.0 | 3.9±3.9 | 3.5±2.3 | 0.21 | 0.32 | 3.9±4.2 | 3.3±1.9 | 3.3±1.8 | 4.2±3.9 | HOMA-IR |
| 0.56 | **0.04** | 0.31±0.02 | 0.32±0.03 | | 0.33±0.03 | 0.33±0.04 | 0.20 | 0.21 | 0.33±0.04 | 0.32±0.02 | 0.32±0.02 | 0.33±0.03 | 0.10 | 0.11 | 0.32±0.03 | 0.32±0.02 | 0.32±0.02 | 0.32±0.04 | QUICKI |
| **0.01** | **0.01** | 119±18 | 121±13 | | 126±16 | 123±14 | 0.45 | **0.04** | 128±14 | 123±19 | 121±15 | 118±14 | 0.14 | 0.18 | 120±20 | 121±14 | 125±14 | 122±13 | SBP (mmHg) |
| 0.24 | **0.01** | 78±13 | 79±11 | | 85±10 | 82±9 | **0.01** | **0.01** | 85±9 | 81±13 | 81±11 | 78±11 | **0.03** | **0.04** | 79±13 | 80±10 | 84±11 | 82±10 | DBP (mmHg) |
| 0.61 | 0.63 | 145±98 | 147±90 | | 148±76 | 162±106 | 0.34 | **0.03** | 171±104 | 157±95 | 132±68 | 141±85 | 0.22 | 0.20 | 143±77 | 153±105 | 166±79 | 138±72 | TG (mg/dl) |
| 0.46 | 0.48 | 187±35 | 193±33 | | 190±40 | 195±38 | 0.85 | 0.87 | 194±40 | 190±37 | 190±35 | 191±33 | 0.17 | 0.25 | 187±32 | 194±36 | 196±35 | 188±41 | TC (mg/dl) |
| 0.35 | 0.37 | 118±31 | 124±31 | | 124±32 | 127±33 | 0.32 | 0.34 | 128±34 | 121±32 | 123±28 | 120±32 | 0.08 | 0.09 | 117±30 | 123±30 | 129±33 | LDL-C (mg/dl) 123±32 | |
| 0.12 | 0.16 | 44±10 | 44±9 | | 43±10 | 43±10 | 0.36 | 0.38 | 43±9 | 42±8 | 44±10 | 44±8 | 0.17 | 0.18 | 43±8 | 44±10 | 41±8 | HDL-C (mg/dl) 45±10 | |

P1-value = P ANOVA test; P2 = ANCOVA test adjusted for sex, age, education, physical activity, marital status, energy intake and BMI. Continuous variables are presented as the means ± standard deviations. *Abbreviations*: DBP, diastolic blood pressure; FBG, fasting blood glucose; HDL-C, high-density lipoprotein cholesterol; HOMA-IR, Homeostasis model assessment-insulin resistance index; QUICKI, quantitative insulin sensitivity check index; SBP, systolic blood pressure; TG, triglyceride; WC, waist circumference; WHR, Waist-to-hip ratio.

**Table 4.** Odd’s ratio (OR) and confidence interval (CI) for MetS and its components according to quartiles (Q) of nutrient patterns in a cross-sectional study of MetS in Iran (n=347)

| Mineral based **p** Simple sugar based **p** Fat based **p** | | | | | | | | | | | | | | | **Variable** |
| --- | --- | --- | --- | --- | --- | --- | --- | --- | --- | --- | --- | --- | --- | --- | --- |
| **Q1** (n=87) **Q2** (n=86)  **Q3** (n=87)  **Q4** (n=87) **Q1** (n=87) **Q2** (n=86)  **Q3** (n=87) **Q4** (n=87) **Q1** (n=86) **Q2** (n=87) **Q3** (n=87)  **Q4** (n=87) | | | | | | | | | | | | | | |  |
|  |  |  |  |  |  |  |  |  |  |  |  |  |  |  | **MetS** ^A^ |
| 0.24 | 1.28(0.82-1.65) | 1.16(075-1.79) | 1.01 (0.65, 1.56) | 1 (Ref.) | 0.74 | 0.79 (0.52-1.18) | 0.86 (0.57-1.29) | 0.89 (0.59-1.33) | 1 (Ref.) | 0.88 | 0.97 (0.65-1.46) | 0.93 (0.62-1.40) | 1.10 (0.73-1.64) | 1 (Ref.) | **Crude** |
| 0.12 | 0.62(0.28-0.91) | 0.56(0.32-0.99) | 0.68 (0.40, 1.14) | 1 (Ref.) | 0.06 | 0.84 (0.54-1.30) | 0.88 (0.57-1.36) | 0.79 (0.52-1.18) | 1 (Ref.) | 0.18 | 1.39(0.85-2.27) | 0.98(0.59-1.59) | 1.09(0.67-1.76) | 1 (Ref.) | **Model 1^B^** |
| 0.36 | 0.60(0.29-0.84) | 0.53(0.28-0.75) | 0.59 (0.43, 1.22) | 1 (Ref.) | 0.62 | 0.77 (0.49-1.20) | 0.78 (0.49-1.23) | 0.78 (0.50-1.22) | 1 (Ref.) | 0.73 | 1.02 (0.65-1.58) | 0.87 (0.56-1.35) | 1.12(0.72-1.72) | 1 (Ref.) | **Model 1^C^** |

P values are reported based on the multivariate multinomial logistic regression test and are considered significant at ˂0.05. The multivariate multinomial logistic regression was used for estimation of ORs and confidence interval (CI). **^A^** Defined as the presence of at least three of the following components: TG ≥150 mg/dl; WC ≥ 88 cm in women and ≥102 cm in men; DBP ≥85 mmHg or SBP ≥ 130 mmHg; HDL-C < 50 mg/dl in women and < 40 mg/dl in men and fasting glucose ≥100 mg/dl. **^B^** Model 1: Adjusted for age, sex, occupation, marital status, education, smoking status, and physical activity. ^C^ Model 2: Model 1 + energy intake. *Abbreviations*: MetS metabolic syndrome
